# Early Clinical Improvement With Lamotrigine Augmentation in Severe Treatment-Resistant Unipolar Depression: A Case Report

**DOI:** 10.7759/cureus.97246

**Published:** 2025-11-19

**Authors:** Nikith Senthilkumar, Rachel Daly

**Affiliations:** 1 Psychiatry, Little Brooke Hospital, Dartford, GBR

**Keywords:** antidepressant augmentation, lamotrigine, phq-9, trd, unipolar depression

## Abstract

Treatment-resistant depression (TRD) is a major clinical challenge, with limited augmentation strategies beyond conventional antidepressants. Lamotrigine, an anticonvulsant licensed for bipolar depression, has been trialled in unipolar TRD, though evidence remains inconsistent, and its use is currently off-license. We describe a 45-year-old gentleman with severe TRD who failed multiple trials of antidepressants, including citalopram and mirtazapine in the community, and subsequently fluoxetine during admission following a suicide attempt. Given his lack of response, lamotrigine augmentation was initiated. Within three weeks, he demonstrated substantial improvement in mood, engagement, and self-care, alongside near-complete resolution of suicidal ideation. Depression severity, measured by the Patient Health Questionnaire-9 (PHQ-9), decreased from 23/27 (severe) to 3/27 (minimal) three weeks post lamotrigine. This rapid response occurred earlier than typically reported in clinical studies. The case highlights lamotrigine as a potentially valuable augmentation option in TRD and emphasises the need for further research to clarify its efficacy and predictors of early response.

## Introduction

Treatment-resistant depression (TRD) is a subset of major depressive disorder characterised by a failure to respond to two or more adequate antidepressant trials. It remains a major clinical challenge and is often associated with poor outcomes, including functional deterioration, persistent chronic symptoms, and an increased risk of suicide.

Current management strategies include dose optimisation, switching antidepressant medications, and combination therapy. In selected cases, augmentation with antipsychotics or mood stabilisers may be beneficial, alongside psychotherapy and neurostimulation techniques [1].

Lamotrigine, primarily an anticonvulsant, has a well-established role in the maintenance and prevention of bipolar depression [2]. Lamotrigine’s use in unipolar depression has not been as extensively studied, and its use in clinical practice is usually off-license [3,4]. Early trials and RCTs prior to 2019 have often reported minimal or inconsistent benefits [5,6]. The 2019 meta-analysis by Goh et al., which included 8 RCTs with 677 participants, found that lamotrigine augmentation produced modest but statistically significant improvements in depressive severity and response rates, particularly among patients with chronic, severe depression or those receiving concurrent selective serotonin reuptake inhibitors (SSRI) therapy [4].

We report a severe case of treatment-resistant depression in which lamotrigine augmentation resulted in marked clinical improvement within three weeks, a substantially faster response than observed in previous studies.

## Case presentation

A 45-year-old man was admitted to the acute male psychiatric unit in early July following ingestion of household bleach with suicidal intent. Approximately four months prior to his admission, he had been experiencing significant workplace bullying, and due to the hostile environment, he chose to leave his job. Following his resignation, he gradually developed pervasive sadness, anhedonia, and suicidal thoughts, in the absence of psychotic symptoms. He did not have any previous history of mental or physical health conditions and was not taking any regular medication. There was no family history of mental health issues either.

He initially sought help from his General Practitioner in early March, who commenced citalopram at 20 mg once daily and subsequently increased the dose to 40 mg over the following weeks. Despite adherence to citalopram for over 10 weeks, he experienced no meaningful improvement in mood, which contributed to increasing feelings of hopelessness.

Whilst on holiday with his partner in mid-June, he urgently took a flight back home and attended Accident & Emergency (A&E) due to severe anxiety and suicidal ideation, to the extent that he felt unsafe to return to his household. At this point, he was open to engage with all forms of treatment and had agreed to informal admission. Due to a combination of limited bed availability and usage of the least restrictive option, he was seen in the community by the Home Treatment Team. Whilst in the community, he was commenced on 15 mg mirtazapine every night. Two weeks later, he ingested the bleach and was admitted as an informal patient.

On arrival to the acute male unit, his mirtazapine was increased to 45 mg, but there was still no improvement. His mental state further deteriorated, with worsening psychomotor retardation, poor engagement, and impaired self-care. He eventually had to be sectioned under the Mental Health Act due to worsening suicidal ideation. Four weeks after admission, his citalopram was stopped, and fluoxetine 20 mg was added and up-titrated to 40 mg (early August). Despite this, no clinical improvement was observed by mid-September, after approximately 5-6 weeks of adherence to fluoxetine 40 mg daily. Given the lack of response to 2 antidepressants at therapeutic doses and durations, the MDT decided to initiate lamotrigine augmentation, starting at 25 mg once daily with weekly titration.

Within 3 weeks, his lamotrigine had been increased to 50 mg twice daily, and he was showing significant improvement in mood, engagement, and self-care. He reported feeling more hopeful and was able to envision a future with his wife, which he had previously been unable to do. His suicidal ideations were nearly non-existent. Although he still experienced a low mood, this was a substantial change that was not achieved with antidepressants alone. There were no adverse side effects of lamotrigine observed.

Depression severity was also measured using the Patient Health Questionnaire-9 (PHQ-9). His score was 23/27 prior to lamotrigine, consistent with severe major depressive episode, and had reduced to 3/27 at 3 weeks post-lamotrigine, consistent with minimal depressive symptoms [7].

## Discussion

This case portrays a rapid response to lamotrigine in a patient with severe unipolar depression. The meta-analysis by Goh et al. reported statistically significant improvements in depressive symptoms, particularly in patients with chronic or severe depression, though with several limitations. The trials included were short and variable in duration (4-12 weeks), had heterogeneous inclusion criteria, and the positive findings were largely driven by studies from China, which may limit generalisability to broader populations. Importantly, there was no clear data on the time for clinical response, making it difficult to establish the typical onset of lamotrigine’s antidepressant effects [4].

Other studies since 2019 report similar methodological constraints such as small sample sizes [8,9]. Despite these limitations, lamotrigine remains a viable augmentation option in the management of TRD, partly due to its favourable side-effect profile compared to other mood stabilisers [10].

In this context, the reduction in PHQ-9 scores from 23/27 (severe) to 3/27 (minimal) within 3 weeks provides strong objective evidence of early improvement. This supports the subjective and behavioural changes observed and suggests lamotrigine may, in some cases, achieve remission earlier than previously reported.

As this report describes only a single patient, the findings cannot be generalised to all individuals with treatment-resistant depression, but it illustrates that early and clinically significant improvement is possible. This case, therefore, adds to the small but growing literature on lamotrigine in unipolar treatment-resistant depression and highlights the need for further high-quality studies to clarify efficacy and identify predictors of response.

## Conclusions

This case demonstrates that lamotrigine augmentation can result in rapid and clinically significant improvement in severe treatment-resistant unipolar depression, as evidenced by both subjective clinical changes and objective reduction in PHQ-9 scores. While conclusions are limited by the single case observation, the findings support considering lamotrigine as an adjunct in select cases and highlight the need for further research to identify predictors of early response.
